# Complete genome sequence of the chromate-reducing bacterium *Thermoanaerobacter thermohydrosulfuricus* strain BSB-33

**DOI:** 10.1186/s40793-015-0028-7

**Published:** 2015-10-05

**Authors:** Pamela Bhattacharya, Adam Barnebey, Marcin Zemla, Lynne Goodwin, Manfred Auer, Steven M. Yannone

**Affiliations:** Life Sciences Division, Lawrence Berkeley National Laboratory, Building 84, Mail Stop 84-171, 1 Cyclotron Road, Berkeley, CA 94720 USA; Bioscience Division, Los Alamos National Laboratory, Los Alamos, NM USA

**Keywords:** *Thermoanaerobacter*, Thermophilic, Anaerobic, Gram-positive, Chromate, Chromium, Reducing, Metal, Bioremediation

## Abstract

*Thermoanaerobacter thermohydrosulfuricus* BSB-33 is a thermophilic gram positive obligate anaerobe isolated from a hot spring in West Bengal, India. Unlike other *T. thermohydrosulfuricus* strains, BSB-33 is able to anaerobically reduce Fe(III) and Cr(VI) optimally at 60 °C. BSB-33 is the first Cr(VI) reducing *T. thermohydrosulfuricus* genome sequenced and of particular interest for bioremediation of environmental chromium contaminations. Here we discuss features of *T. thermohydrosulfuricus* BSB-33 and the unique genetic elements that may account for the peculiar metal reducing properties of this organism. The *T. thermohydrosulfuricus* BSB-33 genome comprises 2597606 bp encoding 2581 protein genes, 12 rRNA, 193 pseudogenes and has a G + C content of 34.20 %. Putative chromate reductases were identified by comparative analyses with other *Thermoanaerobacter* and chromate-reducing bacteria.

## Introduction

*Thermoanaerobacter thermohydrosulfuricus* strain BSB-33 (ATCCBAA-2171 = DSM 25103) is a Gram-positive anaerobic rod-shaped thermophilic bacterium isolated from sediment samples collected at a shallow hot spring in Bakreshwar India in the state of West Bengal [1]. The hot spring sediment was found to be basic (pH 9.2 +/− 0.1) with a temperature range of 66–70 °C supporting a diverse microbial community including *Gammaproteobacteria*, *Cyanobacteria*, green nonsulfur and low-GC Gram-positive bacteria [2]. Strain BSB-33 was found to reduce both Cr(VI) and Fe(III) anaerobically at 60 °C while utilizing peptone or pyruvate [1]. In contrast, *Thermoanaerobacter thermohydrosulfuricus* strain DSM 567^T^ isolated from sugar beet extraction juice, and strain WC1-12 isolated from wood compost are not reported to reduce metals but reduce sulfite and thiosulfate to H_2_S while fermenting a wide range of carbohydrates [3, 4]. The Fe(III) and Cr(VI) reducing trait of BSB-33 has not been reported for the closely related species *Thermoanaerobacter wiegelii* strain Rt8.B1 isolated in New Zealand [5] while *Thermoanaerobacter siderophilus* SR4 isolated from hydrothermal vents in Kamchatka peninsula is capable of reducing Fe(III) only [6]. The related species *Thermoanaerobacter ethanolicus* JW200 and *Thermoanaerobacter pseudethanolicus* 39E isolated from Yellowstone National Park [7, 8] also reduce iron but not chromium [9]. *Thermoanaerobacter* species reported to anaerobically reduce Fe(III) and Cr(VI) (sp X514, sp X513, sp X561) were all collected from the geologically and hydrologically isolated deep subsurface environments in the Piceance Basin in Colorado [10]. Notably however, 16S rRNA and chaperonin-60 universal target (*cpn60* UT) region sequence comparison reveals that the chromate-reducing *Thermoanaerobacter* species (BSB-33, and sp X513-14) are only distantly related. Thus the mechanism(s) of chromium reduction in these two species is likely quite divergent.

Owing to its strong oxidizing nature, soluble Cr(VI) can be toxic, mutagenic, and carcinogenic in various biological systems. Reduction of hexavalent chromium produces the water-insoluble and less mobile Cr(III), which has diminished toxicity due to decreased bioavailability [11]. Chromium (VI) is widely used in many industrial applications and improper disposal of waste has contaminated soil, vadose zones, and groundwater at sites throughout the industrialized world. High levels of Cr(VI) contaminated ground water from leather industries has been reported in South Australia and India [12, 13] and at chromium chemical production sites in Corvalis, Oregon, and Tamil Nadu in India [14, 15]. In addition, the U.S. Department of Energy uranium production and enrichment facilities operating during World War II resulted in high levels of chromium contamination in the groundwater at some U.S. sites [16–18]. Microbe-mediated reduction of soluble Cr(VI) in groundwater into insoluble forms to reduce contamination has been a research pursuit for decades and has been implemented in the U.S. and elsewhere. *Pseudomonas* strains isolated from chromate containing sewage sludge were among the first microbes reported as capable of biological reduction of chromate [19, 20]. In addition, chromium reducing species like *Streptomyces* sp MC1 isolated from sugarcane, and specific microbial soil communities have been used to remediate Cr(VI) contaminated soils [21–23]. Thereafter, several anaerobic chromium reducing bacteria were isolated from chromium-contaminated and non-contaminated soil and ground water and identified as candidates for bioremediation [24]. Despite many advances, there remains much foundational knowledge to gain about the mechanism of bacterial resistance to chromate that will improve our fundamental understanding of microbial metal reduction and improve microbial bioremediation strategies.

Our analyses here reveal that the distinctive chromium-reducing capabilities of BSB-33 appear divergent from other members of this species and may be particularly well-suited for bioremediation of chromium contamination. Here we present a summary classification and a set of features for *Thermoanaerobacter thermohydrosulfuricus* BSB-33 together with the description of the completed genome sequence and annotation. We use this genome sequence for comparative analyses with other chromium reducing bacteria both in and outside of the genus *Thermoanaerobacter*. We report novel insights into the divergent mechanism(s) of chromate reduction and resistance in this highly divergent species.

## Organism information

### Classification and features

**Fig. 1:**
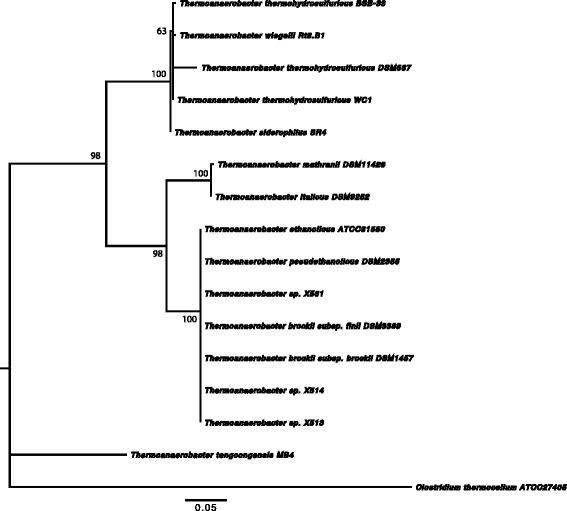
Phylogenetic tree highlighting the position of *Thermoanaerobacter thermohydrosulfuricus* BSB-33 relative to other type strains within the *Thermoanaerobacteraceae*. The tree was inferred from aligned characters of the *cpn60* UT sequences using maximum likelihood method within the software MEGA v5.1 (bootstrap: calculated with the Kimura 2-parameter model distance correction and 1000 replicates). The strains and their corresponding GenBank accession numbers for *cpn60* UT genes are: *T. wiegelii* Rt8.B1, JGI; *T. thermohydrosulfuricus* WC1, HM623896; *T. thermohydrosulfuricus* BSB-33, JGI; *T. thermohydrosulfuricus* DSM567, HM623910; *T. siderophilus* SR4, JGI; *T. mathranii* DSM11426, DQ439966; *T. italicus* DSM9252, NZ_ACVH01000076; *T. ethanolicus* ATCC31550, NZ_ACXY01000003; *T. brockii* subsp. *finii* DSM3389, NZ_ACQZ01000003; *T. brockii* subsp. *brockii* DSM1457, HM623909; *Thermoanaerobacter* sp. X513, ACPF01000040; *Thermoanaerobacter* sp. X514, NC_010320; *T. pseudethanolicus* DSM2355, NC_010321; *Thermoanaerobacter* sp. X561, ACXP01000037; *T. tengcongensis* MB4, NC_003869; *Clostridium thermocellum* ATCC27405, NC_009012. Bootstrap values are indicated at nodes when larger than 60 %. *Clostridium thermocellum* was used as an out group. The branches are scaled in terms of the expected number of substitutions per site (scale bar)

**Fig. 2:**
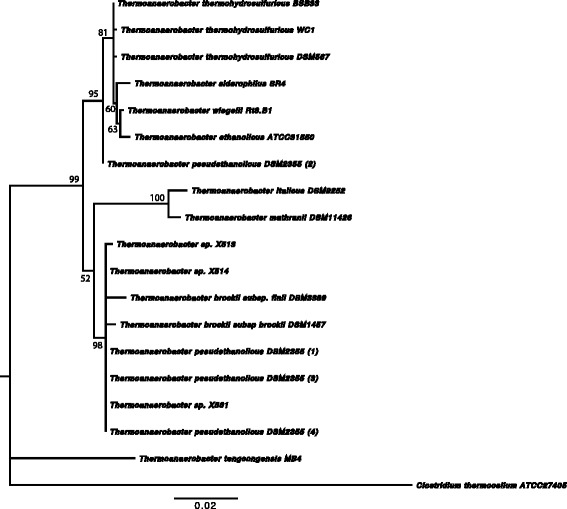
Phylogenetic tree highlighting the position of *Thermoanaerobacter thermohydrosulfuricus* BSB-33 relative to other type strains within the *Thermoanaerobacteraceae*. The tree was inferred from aligned characters of the 16S rRNA sequences using maximum likelihood method within the software MEGA v5.1 (bootstrap: calculated with the Kimura 2-parameter model distance correction and 1000 replicates). The strains and their corresponding GenBank accession numbers for 16S rDNA respectively are: *T. wiegelii* Rt8.B1, NR_075059.1; *T. thermohydrosulfuricus* WC1, HM585213.1; *T. thermohydrosulfuricus* BSB-33, EU368841.2; *T. thermohydrosulfuricus* DSM567, L09161.1; *T. siderophilus* SR4, AF120479.1; *T. mathranii* DSM11426, NR_026397.1; *T. italicus* DSM9252, AJ250846.1; *T. ethanolicus* ATCC31550, L09162.1; *T. brockii* subsp. *finii* DSM3389, L09166.1; *T. brockii* subsp. *brockii* DSM1457, L09165.1; *Thermoanaerobacter* sp. X513, AF542520.1; *Thermoanaerobacter* sp. X514, AF542517.1; *T. pseudethanolicus* DSM2355, L09164; *Thermoanaerobacter* sp. X561, AF542518.1; *T. tengcongensis* MB4, AF209708.1; *Clostridium thermocellum* ATCC27405, L09173.1. Bootstrap values are indicated at nodes when larger than 60 %. *Clostridium thermocellum* was used as an out group. The branches are scaled in terms of the expected number of substitutions per site (scale bar)

Strain BSB-33 has been previously described as ‘*Thermoanaerobacter*-like bacterium’ [1] on the basis of BLAST [25] analysis of a 968 bp 16S rRNA sequence (EU368841) obtained using a single S-D-Bact-0027-a-S-18 primer [26]. Later, BSB-33 16S rRNA gene was re-sequenced by the American Type Culture Collection to generate a 1614 bp long sequence that has been deposited in GenBank (EU368841.2). The BSB-33 strain is deposited in the ATCC as ‘*Thermoanaerobacter**indiensis* BSB-33’. However, comparison of the 16S rRNA sequence using NCBI BLAST shows that BSB-33 has >99 % 16S rRNA sequence identity with *Thermoanaerobacter thermohydrosulfuricus* DSM 567, *Thermoanaerobacter thermohydrosulfuricus* WC1, *Thermoanaerobacter wiegelii* Rt8.B1, *Thermoanaerobacter pseudethanolicus* ATCC 33223, and *Thermoanaerobacter siderophilus* SR4. Thus ‘*Thermoanaerobacter**indiensis* BSB-33’ cannot be identified as a unique species on the basis of 16S rRNA sequence. Notably however, taxonomic assignment of *Thermoanaerobacter* species using 16S rRNA sequence is known to be particularly problematic due to highly conserved intervening sequences (IVS) in 16S rRNA of *T. thermohydrosulfuricus* and *T. pseudethanolicus* [4]. Phylogenetic analysis for identification of *Thermoananerobacter* species based on chaperonin-60 universal target region proved more accurate than 16S rRNA sequence and a more accurate predictor of whole genome relatedness and DNA-DNA hybridization values [27]. Therefore, phylogenetic trees have been constructed for BSB-33 using the maximum likelihood (ML) method within MEGA v5.1 software [28]. We used both chaperonin-60 universal target region and 16S rDNA derived from sequenced *Thermoanaerobacter* genomes to compare the trees and *Clostridium thermocellum**was used* to root the trees (Figs. 1 and 2). Despite apparent phenotypic divergence, BSB-33 exhibited 99.82 %, 99.64 %, 99.64 %, 98.19 % chaperonin-60 UT sequence identity with *T. thermohydrosulfuricus* WC1, *T. wiegelii* Rt8.B1, *T. siderophilus* SR4, and *T. thermohydrosulfuricus* DSM 567 respectively. The 16S rRNA sequence identity of BSB-33 with *T. thermohydrosulfuricus* WC1, *T. wiegelii* Rt8.B1, *T. siderophilus* SR4 and *T. thermohydrosulfuricus* DSM 567 is further supported by sequence identity of *cpn60* UT region; hence, it is phylogenetically closer with these species than with other *Thermoanaerobacter* species represented in these analyses. Despite significant genotypic and phenotypic diversity across *Thermoanaerobacter* sub-species, genetic homology in standard marker genes results in classification of diverse strains as a common species [4]. Because of this difficulty with *Thermoanaerobacter* species, and to infer whole genome relatedness, digital DNA-DNA hybridization by means of genome-to-genome distances (GGD) were calculated using the GGDC 2 software [29]. The BSB-33 genome (KB910517.1) shares 95.2 %, 84.8 % and 76 % DDH identity with *T. thermohydrosulfuricus* WC1 (KB731262.1 – KB731323.1), *T. siderophilus* SR4 (CM001486.1) and *T. wiegelii* Rt8.B1 (CP002991.1) genomes respectively. The typical cutoff for a unique species is a genome relatedness of less than 70 % [30]. Since BSB-33 shares the greatest sequence identity with WC1, it is therefore most likely that ‘*Thermoanaerobacter**indiensis* BSB-33’ is a divergent subspecies of *T. thermohydrosulfuricus* and hereafter referred to as strain BSB-33.

**Fig. 3:**
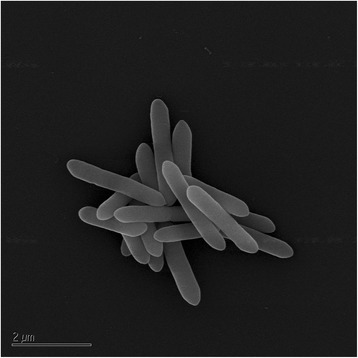
Scanning electron micrograph of *T. thermohydrosulfuricus* BSB-33 grown anaerobically in nitrogen-purged Luria-Bertani (LB) broth at 60 °C to mid exponential phase

BSB-33 cells are described as straight to curved rods, approximately 0.5 μm in diameter and 2 μm in length occurring singly or in short filaments (Fig. 3) [1]. Strain BSB-33 was determined to be gram-positive at all stages of growth with slight tumbling motility. Colonies were uniformly round, white, opaque and 0.5-0.8 mm in diameter when grown anaerobically for 2–3 days on 2 % agar plates (not shown). The G + C content of the DNA of strain BSB-33 was determined by the thermal denaturation to be 35.70 ± 2 mol% [1], and calculated to be 34.20% from the genome sequence reported here (see Table 3).

BSB-33 cells are strict anaerobes that can couple the oxidation of peptone to the reduction of Fe(III) oxyhydroxide and Fe(III)-citrate with maximal growth and iron and chromium reduction observed at 60 °C [1]. In basal medium BSB-33 can also couple the oxidation of peptone to the reduction of 0.2 mM K_2_CrO_4,_ 30 mM MnO_2_ and 20 mM AQDS [1]. When Fe (III)-citrate is used as the electron acceptor either peptone or pyruvate (organic acid) can be utilized as a substrate (electron donor) while lactate, acetate, citrate, or 100 % hydrogen gas are not utilized [1]. Both the soluble and membrane subcellular fractions of BSB-33 contain anaerobic Fe(III) and Cr(VI) reduction activities at 60 °C using NADH as the electron donor [1]. A summary of the classification and general features of *T. thermohydrosulfuricus* BSB-33 are presented in Table 1.

**Table 1:** Classification and general features of Thermoanaerobacter thermohydrosulfuricus BSB-33 according to MIGS recommendations [66]

MIGS ID	Property	Term	^a^Evidence code
	Classification	Domain *Bacteria*	TAS [67]
		Phylum *Firmicutes*	TAS [68–70]
		Class *Clostridia*	TAS [71, 72]
		Order *Thermoanaerobacterales*	TAS [71, 73]
		Family *Thermoanaerobacteraceae*	TAS [71, 74]
		Genus *Thermoanaerobacter*	TAS [1, 75]
		Species *Thermoanaerobacter thermohydrosulfuricus*	TAS [3]
	Gram stain	Positive	TAS [1]
	Cell shape	Rod-Shape	TAS [1]
	Motility	Slight tumbling motility	TAS [1]
	Sporulation	Not reported	
	Temperature range	Thermophile	TAS [1]
	Optimum temperature	60 °C	TAS [1]
	pH range; Optimum	Not reported	
	Carbon source	Pyruvate	TAS [1]
MIGS-6	Habitat	Hot spring, Aquatic, Marine	TAS [1]
MIGS-6.3	Salinity	Not reported	
MIGS-22	Oxygen requirement	Obligately anaerobe	TAS [1]
MIGS-15	Biotic relationship	Free living	TAS [1]
MIGS-14	Pathogenicity	Non pathogenic	TAS [1]
MIGS-4	Geographic location	West Bengal, India	TAS [1]
MIGS-5	Sample collection	Not reported	
MIGS-4.1	Latitude	23.52	NAS
MIGS-4.2	Longitude	87.22	NAS
MIGS-4.3	Depth	Not reported	
MIGS-4.4	Altitude	84 m	NAS

^a^Evidence codes - IDA: Inferred from Direct Assay; TAS: Traceable Author Statement (*i.e.*, a direct report exists in the literature); NAS: Non-traceable Author Statement (*i.e.*, not directly observed for the living, isolated sample, but based on a generally accepted property for the species, or anecdotal evidence). These evidence codes are from the Gene Ontology project [76]

## Genome sequencing information

### Genome project history

BSB-33 was selected for sequencing by the DOE Joint Genome Institute Community Sequencing Program 2012 because of its phylogenetic position and its metal reducing capabilities that are potentially suitable for DOE missions involving bioremediation and microbial fuel cell development. The genome project is deposited in Integrated Microbial Genome (IMG ER) online database in the Genome Online Database GOLD [31] as project Gi14051 and the complete genome sequence is available in GenBank (KB910517.1) under the name ‘*Thermoanaerobacter**indiensis* BSB-33’. Sequencing, finishing, and annotation of *T. thermohydrosulfuricus* BSB-33 were performed by DOE Joint Genome Institute. A summary of the project information is shown in Table 2.

**Table 2:** Project information

MIGS ID	Property	Term
MIGS-31	Finishing quality	Improved high-quality draft
MIGS-28	Libraries used	Illumina Standard (short paired-end)/Illumina CLIP (long paired-end) library
MIGS-29	Sequencing platform	Illumina
MIGS-31.2	Fold coverage	3082X
MIGS-30	Assemblers	Allpaths r41554/Velvet 1.1.05/Parallel phrap 1.1.05
MIGS-32	Gene calling method	Prodigal 1.4, GenePRIMP
	Locus Tag	B044
	Genbank ID	KB910517.1
	Gene bank date of release	April 16, 2013
	GOLD ID	Gi14051
	BioProject	PRJNA169716
MIGS-13	Source material identifier	DSM 251035
	Project relevance	Chromium bioremediation

### Growth conditions and genomic DNA preparation

*T. thermohydrosulfuricus* BSB-33 was grown anaerobically in nitrogen (N_2_) purged Luria-Bertani (LB) broth at 60 °C. Extraction of chromosomal DNA was performed on 500 ml of culture grown into late exponential phase (OD_600nm_ = 0.4). Cells were collected by centrifugation at 4 °C and 10,000x g for 10 min. High molecular weight DNA was prepared from these cells using the standard methods as recommended by the DOE Joint Genome Institute (JGI, Walnut Creek, CA, USA). Briefly, cells were suspended in TE buffer (10 mM Tris–HCl, 1.0 mM EDTA, pH 8.0) to a final OD_600nm_ = 1. The sample was treated with lysozyme (1.33 mg/ml) and incubated 5 min at room temperature. Sodium dodecyl sulfate (0.5 %) and Proteinase K (0.1 mg/ml) were added and cells incubated for 1 hr at 37 °C. NaCl (0.5 M) and hexadecyltrimethyl ammonium bromide/NaCl mixture (0.66 %/46 mM) were added and incubated at 65 °C for 10 min and then extracted with chloroform/isoamyl alcohol mixture. Cell lysates were extracted with phenol/chloroform/isoamylalcohol (25:24:1) followed by precipitation with 0.6x volumes of isopropanol. The nucleic acid pellet was washed with 70 % ethanol, dissolved in TE buffer and then treated with DNase-free RNase A at 37 °C for 20 min. DNA concentration and purity was determined by UV/Vis absorbance and DNA quality further assessed by visualizationon 1 % agarose gels stained with ethidium bromide and no plasmid or viral DNA was evident in preparations (not shown).

### Genome sequencing and assembly

The draft genome of *Thermoanaerobacter thermohydrosulfuricus* BSB-33 (*‘T. indiensis’* BSB-33 in JGI documents) was generated at the DOE Joint Genome Institute (JGI) using Illumina data [32]. For this genome, a short-insert paired-end library was constructed and sequenced with Illumina giving average insert sizes of 270 +/− 70 bp which generated 26,606,974 reads and an Illumina long-insert paired-end library gave an average insert size of 10871 +/− 1786 bp which generated 40,206,744 reads totalling 8,012 Mbp of Illumina data (unpublished, Feng Chen-JGI). All general aspects of library construction and sequencing performed at the JGI can be found at http://www.jgi.doe.gov/. The initial draft assembly contained 41 contigs in ten scaffold(s). The initial draft data was assembled with Allpaths, version r41554 [33], and the consensus was computationally shredded into 10 Kbp overlapping fake reads (shreds). The Illumina draft data was also assembled with Velvet, version 1.1.05 [34] and the consensus sequences were computationally shredded into 1.5 Kbp overlapping fake reads (shreds). The Illumina draft data was assembled again with Velvet using the shreds from the first Velvet assembly to guide the next assembly. The consensus from the second Velvet assembly was shredded into 1.5 Kbp overlapping fake reads. The fake reads from the Allpaths assembly and both Velvet assemblies and a subset of the Illumina CLIP paired-end reads were assembled using parallel phrap, version 4.24 (High Performance Software, LLC). Possible mis-assemblies were corrected with manual editing in Consed [35–37]. Gap closure was accomplished using repeat resolution software (unpublished, Wei Gu-JGI), and sequencing of bridging PCR fragments with Sanger and/or PacBio (unpublished, Cliff Han-JGI) technologies. For improved high quality draft and noncontiguous finished projects, one round of manual/wet lab finishing may have been completed. Primer walks, shatter libraries, and/or subsequent PCR reads may also be included for a finished project. A total of zero additional sequencing reactions, 11 PCR PacBio consensus sequences, and 0 shatter libraries were completed to close gaps and to raise the quality of the final sequence. The total size of the genome is 2.6 Mb and the final assembly is based on 8,012 Mbp of Illumina draft data, which provides an average 3,082X coverage of the genome.

### Genome annotation

Genes were identified using Prodigal [38] as part of the Oak Ridge National Laboratory genome annotation pipeline, followed by a round of manual curation using GenePRIMP gene prediction software [39]. The predicted CDSs were translated and used to search the National Center for Biotechnology Information (NCBI) nonredundant database, UniProt, TIGRFam, Pfam, PRIAM, KEGG, COG, and InterPro databases. Additional gene prediction analysis and functional annotation was performed within the Integrated Microbial Genomes (IMG-ER) platform (http://img.jgi.doe.gov) developed by the Joint Genome Institute, Walnut Creek, CA, USA [40].

## Genome properties

**Fig. 4:**
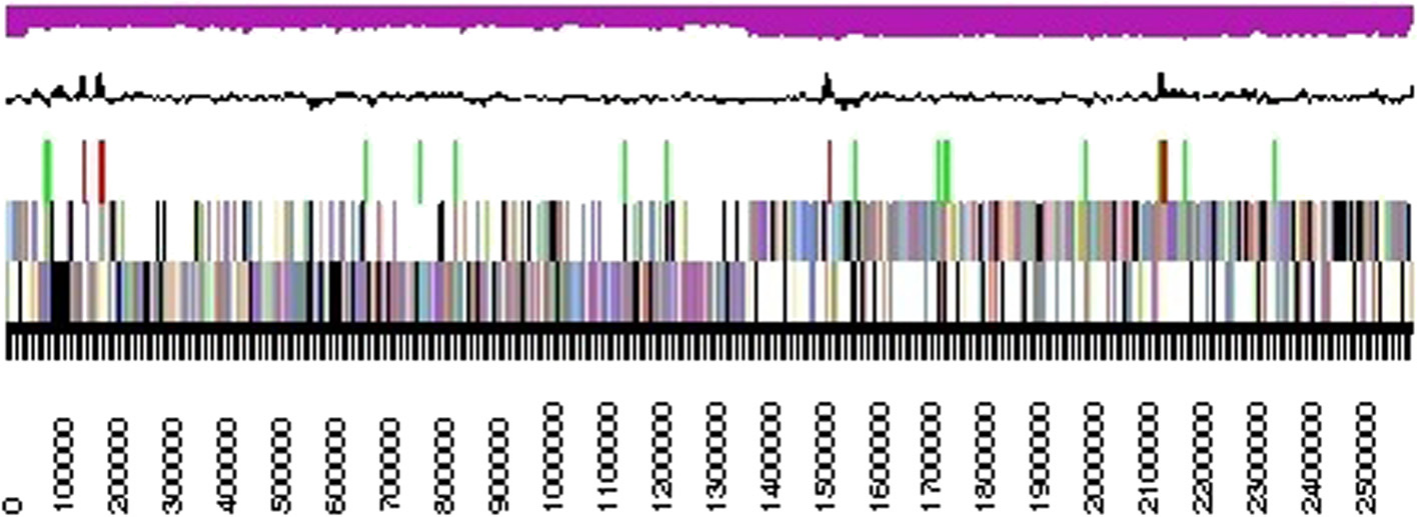
A graphical overview of the *T. thermohydrosulfuricus* BSB-33 genome. The nucleotide numbers are shown across the bottom of the figure with a corresponding black and white scale bar. The first row above the scale bar identifies genes on the forward strand of the genome, color coded according to COG categories. The second row above the scale bar is also color coded by COG category and shows genes on the opposite strand (reverse strand) of the genome. The third row of colored tick marks shows the location of identified RNA genes with tRNAs in green, rRNAs in red. The black line above the protein and RNA gene markers shows the GC content across the genome, and the top magenta colored graphical corresponds to the GC skew across the genome

Detailed genome statistics are provided in Table 3 and Fig. 4. The genome consists of one chromosome with a total length of 2,597,606 bp and a G + C content of 34.20 %. Of the 2,721 predicted genes, 2,581 are protein-coding genes, 140 encode putative RNAs, and 193 pseudogenes were identified. The majority of the protein-coding genes (79.86 %) were assigned a putative function while the remaining genes were annotated as hypothetical proteins. The distribution of genes into functional categories based on clusters of orthologous genes is presented in Table 4.

**Table 3:** Genome statistics

Attribute	Genome (Total)	
	Value	% of total^a^
Genome size (bp)	2,597,606	100.00
DNA coding sequence (bp)	2,236,208	86.09
DNA G + C content (bp)	888,278	34.20
Number of replicon	1	
Extrachromosomal elements	0	
Total genes	2,721	100.0
RNA genes	140	5.15
rRNA genes	12	0.44
Protein coding genes	2,581	94.85
Pseudo Genes	193	7.09
Genes with function prediction	2,173	79.86
Protein coding genes with COGs	1,759	76.92
Protein coding genes with Pfam	2,244	82.47
Genes in paralog cluster	974	35.8
Protein coding genes coding signal peptides	55	2.02
Protein coding genes coding transmembrane proteins	645	23.7
Genes connected to transporter classification	319	11.72
CRISPR count	5	

^a^The total is based on either the size of the genome in base pairs or the total number of protein coding genes in the annotated genome

**Table 4:** Number of genes associated with general COG functional categories

Code	Value	% of total^a^	Description
J	146	7.58	Translation, ribosomal structure and biogenesis
A	-	-	RNA processing and modification
K	137	7.11	Transcription
L	101	5.24	Replication, recombination and repair
B	-	-	Chromosome structure and dynamics
D	36	1.37	Cell cycle control, cell division, chromosome partitioning
Y	-	-	Nuclear structure
V	33	1.71	Defense mechanisms
T	75	3.89	Signal transduction mechanisms
M	89	4.62	Cell wall/membrane/envelope biogenesis
N	44	2.28	Cell motility
Z	-	-	Cytoskeleton
W	-	-	Extracellular structure
U	37	1.92	Intracellular trafficking, secretion and vesicle transport
O	65	3.37	Posttranslational modification, protein turnover
C	115	5.97	Energy production and conversion
G	194	10.07	Carbohydrate transport and metabolism
E	182	9.45	Amino acid transport and metabolism
F	57	2.96	Nucleotide transport and metabolism
H	83	4.31	Coenzyme transport and metabolism
I	42	2.18	Lipid transport and metabolism
P	91	4.72	Inorganic ion transport and metabolism
Q	17	0.88	Secondary metabolites biosynthesis, transport and catabolism
R	206	10.7	General function prediction only
S	176	9.14	Function unknown
-	962	35.35	Not in COGs

^a^The total is based on the total number of protein coding genes in the annotated genome

## Insights from the genome sequence

### Chromate reductases

Chromium contamination is primarily an anthropogenic event and therefore microbial chromium reduction may not have specifically evolved for chromate, it is more likely part of generalized electron transport systems or enzymes with other primary physiological functions [41–45]. A number of both soluble and membrane associated chromate-reducing enzymes have been purified to various degrees, most of which have NAD(P)H dependant oxidoreductase activity [44]. Some of these enzymes have proven to be essential for both Cr(VI) reduction and countering chromate mediated oxidative stress, thereby conferring bacterial chromate resistance [41]. Many *Thermoanaerobacter* are capable of reducing iron, but strain BSB-33 has both Fe(III) and Cr(VI) reductase activity, suggesting genotypic dissimilarity [1].

**Fig. 5:**
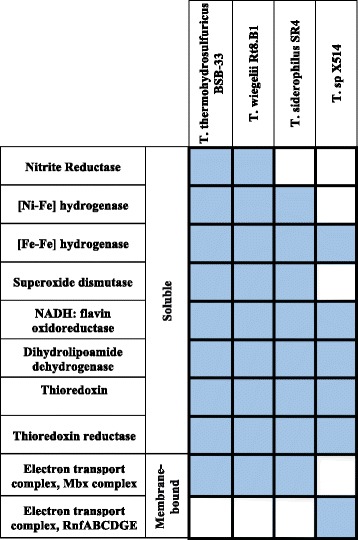
Comparison of candidate chromate reductase genes in *Thermoanaerobacter thermohydrosulfuricus* BSB-33, *T. wiegelii* Rt8.B1, *T. siderophilus* SR4 and *T.* sp X514 species. The grid is arranged with columns representing *Thermoanaerobacter* species and rows representing candidate chromate reductase genes. Each grid is colored depending on whether the gene is present (blue) or absent (white)

To gain insight into the mechanism(s) of chromate reduction and chromate tolerance in BSB-33, comparative genomic analysis was conducted on BSB-33 and its closely related species *T. wiegelii* Rt8.B1 and *T. siderophilus* SR4, and the Fe(III) and Cr(VI) reducing *Thermoanaerobacter* sp X514 isolate. Gene annotations and computational tools available in the Joint Genome Institute Integrated Microbial Genomes database were used for analyses. Independent searches for Cluster of Orthologous Groups [46] and analyzing associated enzyme commission number, KEGG Orthology [47], and TIGRFAMs [48] annotations together with literature and database searches, we identified genes potentially encoding soluble and membrane associated oxidoreductases and dehydrogenases that potentially function in chromate reduction. Genes encoding dihydrolipoamide dehydrogenase, NADH:flavin oxidoreductase (Old yellow enzyme), di-iron [Fe-Fe] hydrogenase, thioredoxin, thioredoxin reductase were found to be potential Cr-reducing proteins common to all four *Thermoanaerobacter* species in our analysis. Interestingly however, NAD(P)H-nitrite reductase, NADH:ubiquinone oxidoreductase (H(+) translocating) complex, nickel-iron [Ni-Fe] hydrogenase, superoxide dismutase were all limited to BSB-33 and its most closely related species *T. wiegelii* Rt8.B1 and *T. siderophilus* SR4 (Table 5). Therefore, differences are expected in the mechanism of chromate reduction by BSB-33 and sp X514. Summary of all features is presented in Fig. 5.

**Table 5:** Candidate chromate reductase genes from Thermoanaerobacter isolates BSB-33, Rt8.B1, SR4 and sp.X514

Enzyme	Locus tag/locus				Molecular function
	*T. thermohydrosulfuricus* BSB-33	*T. wiegelii* Rt8.B1	*T. siderophilus* SR4	*T.* sp. X514	
	Metals reduced – Fe(III), Cr(VI), Mn(IV).	Metals reduced - Not reported	Metals reduced - Fe(III)	Metals reduced – Fe(III), Co(III), Cr(VI), Mn(IV), U(VI)	
Soluble					
Nitrite reductase	B044DRAFT_0957	Thewi_0872			Nitrite reduction
[Ni-Fe] hydrogenase	B044DRAFT_0240	Thewi_0045	WP_039929512.1		H_2_ production, oxidoreductase activity
[Fe-Fe] hydrogenase	B044DRAFT_1055, 1062	Thewi_0980, 1985	WP_006569903.1	Teth514_2138	H_2_ production, oxidoreductase activity
Superoxide dismutase	B044DRAFT_1026	Thewi_0942	WP_006570221.1		Decomposition of O_2_∙^−^
NADH: flavin oxidoreductase (Old yellow enzyme)	B044DRAFT_0057, 0449, 1188	Thewi_0012, 0323, 1077, 1542, 2612	WP_006569792.1, WP_003869984.1, WP_006570529.1	Teth514_0011, 0146, 1378	FMN binding, Oxidoreductase activity
Dihydrolipoamide dehydrogenase	B044DRAFT_0196, 0424	Thewi_0096, 0296,	WP_006569279.1, WP_006569885.1, WP_039929255.1	Teth514_0234, 2038	FAD binding, oxidoreductase activity
Thioredoxin	B044DRAFT_1246	Thewi_1131, 1183	WP_006570115.1	Teth514_1436, 1476	Iron ion binding, Iron sulfur cluster binding
Thioredoxin reductase	B044DRAFT_1204	Thewi_2202	WP_003870064.1	Teth514_0924	oxidoreductase activity
Membrane bound					
Electron transport complex, Mbx (H(+) translocating) complex	B044DRAFT_0131-B044DRAFT_0143	Thewi_0034-Thewi_0046	WP_006569860.1-WP_006569862.1, WP_006569864.1, WP_003869957.1- WP_003869960.1, WP_003869962.1- WP_003869964.1, WP_004399468.1, WP_039929512.1,		Oxidoreductase activity
Electron transport complex, RnfABCDGE Na(+) translocating				Teth514_0079 - Teth514_0084	Electron carrier activity and oxidoreductase activity

### Candidate genes for soluble metal (chromate) reduction activities

#### Nitrite reductases

The formate-dependent nitrite reductase (*nrfA*) in *Shewanella oneidensis* MR1 is involved in nitrite reduction during anaerobic respiration and also proposed to be responsible for chromate reduction [45]. Recently, the formate-dependent cytochrome c nitrite reductase (*nrfA*) was shown to catalyze the six electron reduction of nitrite (NO_2_^−^) to ammonium (NH_4_^+^) at the active site heme iron which also catalyzes sulfite reduction [49]. The BSB-33 genome encodes a NAD(P)H-nitrite reductase (*nirB*) (B044DRAFT_0957; EC:1.7.1.4; COG1251; KO:K00362), which is an iron-sulfur heme flavoprotein containing siroheme which catalyzes the reduction of nitrite to ammonium (N0_2_^−^ + 6[H**∙**] = NH_4_^**+**^+ 20H^−^). The siroheme prosthetic group bound to *nirB* gene products are covalently linked to iron-sulfur clusters which facilitate the six electron reduction of nitrite and sulfite [50, 51]. Owing to such electron transfer characteristics, it is possible that the NAD(H)-nitrite reductases of BSB-33 are also involved in Cr(VI) reduction under anaerobic conditions. The genes encoding enzymes involved in the biosynthesis of siroheme, are *hemABCDL* (B044DRAFT_0544, 0547, 0545, 0546, 0551) to convert glutamyl tRNA to uroporphyrinogen-III, *cobA-hemD* (B044DRAFT_0546) and *MET8* (B044DRAFT_0548) to convert uroporphyrinogen–III to siroheme. These genes are only present in the BSB-33 genome and its most closely related species *T. wiegelii* Rt8.B1 and *T. siderophilus* SR4.

#### Hydrogenases

Di-iron (Fe-Fe) hydrogenase that couple oxidation of NADH and ferrodoxins simultaneously to produce hydrogen gas (H_2_) are conserved within the *Thermoanaerobacter* genus [52]. A six-gene cluster encoding a membrane bound nickel-iron energy conserving hydrogenase (*Ech*) has been identified in the *Thermoanaerobacter* strains *T. italicus* Ab9, *T. mathrani subsp mathrani* A3, *T. wiegelii* Rt8.B1, *T. siderophilus* SR4, and *T. thermohydrosulfuricus* WC1 [52]. The *Ech* complex uses reduced ferredoxins generated by pyruvate catabolism for the evolution of H_2_ [52]. BSB-33 genome contains genes encoding two di-iron (Fe-Fe) hydrogenases (B044DRAFT_1055,1062,) and a nickel-iron (Ni-Fe) hydrogenase (B044DRAFT_0240) in addition to other genes of the Ech complex (B044DRAFT_0236-B044DRAFT_0241). By KEGG orthology (KO) annotation the hydrogenase genes are designated as NADH-quinone oxidoreductase subunit G (KO:K00336) and NADH-quinone oxidoreductase subunit D (KO:K00333) respectively. Based on Enzyme Commission (EC) number, the hydrogenases are assigned (EC:1.6.5.3), which is NADH:ubiquinone reductase (H(+)-translocating), a respiratory chain flavoprotein (FMN) containing iron sulfur clusters and involved in electron transfer from NADH to ubiquinone coupled to transmembrane proton translocation. Soluble bacterial quinone oxidoreductases are thought to reduce Fe(III) and Cr(VI) and counter oxidative stress [53]. The quinone oxidoreductases prevent formation of potentially toxic semiquinone radicals and reactive oxygen species [42]. One of the two di-iron hydrogenase genes in BSB-33 is disrupted by insertion of repetitive elements (B044DRAFT_1062). Therefore, the remaining intact di-iron hydrogenase and the Ni-Fe hydrogenase, which is in the cytoplasmic part of Ech complex, remain candidates for Cr(VI) reduction and oxidative stress responses.

#### Superoxide dismutases

In contrast to *Thermoanaerobacter* sp X514 genome, BSB-33, Rt8.B1 and SR4 genomes encode a superoxide dismutase *SOD*, an antioxidant protein involved in dismutation of superoxide into oxygen and hydrogen peroxide [53]. Reduction of Cr(VI) compounds can give rise to reactive oxygen species which elicit bacterial cell responses that include inducing antioxidant proteins like superoxide dismutases [54]. It has been proposed that improving the efficacy of enzymes to minimize oxidative stress during chromate reduction is one possible way to increase the effectiveness of bioremediation by a bacterial species [54]. Unlike sp X514, the BSB-33 genome contains an additional enzyme (*SOD2,* B044DRAFT_1026) to counter oxidative stress produced during chromate reduction.

#### NADH: flavin oxidoreductase (Old Yellow Enzyme)

The NADH:flavin oxidoreductase from *Thermus scotoductus* SA-01 is related to the Old Yellow Enzyme family and exhibits chromate reductase activity [55]. This chromate-reducing OYE is highly conserved in *Bacillus subtilis* where it is functional in xenobiotic degradation and oxidative stress responses [55]. Likewise, oxidative stress responses in *Azotobacter vinelandii* are linked to flavin-containing oxidoreductase enzymes [56]. OYE-related NADH: flavin oxidoreductases are present in all four *Thermoanaerobacter* species in our analysis. BSB-33 genome encodes three different OYE-related NADH: flavin oxidoreductases (B044DRAFT_0449, 0057, 1188). The NADH: flavin oxidoreductases of BSB-33 (B044DRAFT_0057) and sp X514 (Teth514_0011) share 48 % amino acid sequence identity with the *T. scotoductus* SA-01 OYE homologue. Hence it is possible that one or more of the BSB-33 NADH:flavin oxidoreductases are also involved in chromate reduction and oxidative stress responses.

### Dihydrolipoamide dehydrogenase

Dihydrolipoamide dehydrogenase is part of a multisubunit pyruvate dehydrogenase complex. This complex catalyzes the conversion of pyruvate to acetyl-CoA, and also exhibits chromate reductase activity in *Thermus**Scotoductus* SA-01 [57]. LPD (EC:1.8.1.4) is a flavoprotein belonging to family of pyridine nucleotide-disulfide oxidoreductases (class I active site) [57, 58] and genes encoding LPD have been identified in BSB-33, Rt8.B1, SR4 and sp X514. BLAST analysis reveals that LPD of BSB-33 (B044DRAFT_0196, 0424) shares 36 % and 37 % amino acid identity with *T. scotoductus* SA-01 LPD respectively. LPD protein sequences from sp X514 (Teth514_0234, 2038) share 93 % and 43 % sequence identity with BSB-33 LPD (B044DRAFT_0196). Hence, the LPD of BSB-33 and sp X514 may also be involved in the observed chromate reduction.

#### Thioredoxin and thioredoxin reductase

A metal reduction operon (*mre*) identified in *Desulfovibrio desulfuricans* G20 encoding thioredoxin, thioredoxin reductase and an additional metal oxidoreductase exhibited Cr(VI) and U(VI) reduction [59]. Cr(VI)-exposed cultures of *Caulobacter crescentus* and *Shewanella oneidensis* MR1 showed upregulation of genes encoding thioredoxin and glutaredoxin [44, 60, 61]. BSB-33, Rt8.B1, SR4 and sp X514 genomes encode thioredoxin and thioredoxin reductases. Thus, thioredoxin and thioredoxin reductases in BSB-33 and sp X514 must be formally considered as candidate soluble proteins for chromate reduction by BSB-33.

### Candidate genes for membrane associated chromate reduction activities

#### Mbx and Rnf complex

A 13 gene-cluster similar in genomic structure to membrane bound oxidoreductase (*mbx*) genes of *Pyrococcus furiosus* was identified in three *Thermoanaerobacter* strains, *T. wiegelii* Rt8.B1, *T. siderophilus* SR4 and *T. thermohydrosulfuricus* WC1 [52]. The gene cluster encodes a complex with Fd_red_:NAD(P)^+^ oxidoreductase activity where energy is conserved by translocation of cations with oxidation of ferredoxin (Fd_red_) [52]. BSB-33 is closely related to *T. wiegelii* Rt8.B1 and *T. siderophilus* SR4 and also encodes a similar *mbx* gene cluster (B044DRAFT_0131-B044DRAFT_0143). Six of the *mbx* cluster genes in BSB-33 encode the formate hydrogenlyases subunits 3, 4, 6, and a NADH-quinone oxidoreductases subunit B, and the 27 kDa, and 49 kDa subunits (B044DRAFT_0138-B044DRAFT_0143) that are annotated as NADH-quinone oxidoreductases, flavoproteins (FMNs) containing iron sulfur clusters. Among these formate hydrogenlyase subunit 3,4,6 (B044DRAFT_0138, 0139, 0143) and NADH-quinone oxidoreductases 49KD subunit (B044DRAFT_0142) have transmembrane helices. In *Shewanella putrefaciens* MR1, inhibitory studies suggested that cytoplasmic membrane bound multi-component electron transport chains including cytochromes, quinones, flavoproteins and proteins with iron sulfur centres are involved in Cr(VI) reduction [62]. Therefore, the formate hydrogenlyases and NADH-quinone oxidoreductases 49 kDa subunit of the *mbx* complex in BSB-33 might play an essential role in transferring reducing equivalents to extracellular Cr(VI) ions. In sp X514 all of the 13 genes required for a functional *mbx* complex could not be identified [52]. Instead a functionally analogous Na^+^ ion translocating Rnf complex is present [52]. The Rnf gene cluster encodes for NADH:ubiquinone oxidoreductases (COG4656, TIGR01945) which are possibly involved in Cr(VI) reduction in sp X514.

## Conclusions

*‘T. indensis* BSB-33’ has been classified as *T. thermohydrosulfuricus* BSB-33 based on 16S and *cpn60* UT region sequence identity. Within a given *Thermoanaerobacter* species there is a notedly broad functional diversity with only genetic microdiversity, even among isolates from a common environment [4]. Here we focus on the divergent metal reducing capabilities among members of the *Thermoanaerobacter* genus. Despite many years of intensive study, assimilatory and dissimilatory metal reduction processes in microbes remains incompletely understood and particularly difficult to discern from genetic sequence alone [63, 64]. We present genomic analyses between *Thermoanaerobacter* species to highlight the mechanisms of dissimilatory metal reduction of Cr(IV) and Fe(III) in these microbes. This comparitive genome analysis indicates several oxidoreductases in BSB-33 that are likely to be involved in chromate reduction of which nitrite reductase, dihydrolipoamide dehydrogenase and NADH: flavin oxidoreductase are top candidate genes. These enzymes being redox proteins with flavin and iron sulfur center prosthetic groups which play essential roles in electron transfer have appropriate characteristics to transfer electrons to Cr(VI) [50, 65].

The complete genome sequence of BSB-33 provides the starting point for a detailed analysis of the mechanism of chromate reduction. Novel mechanisms and uncommon dissimilatory metal reduction pathways between *Thermoanaerobacter* strains can be identified by further comparative genomic analysis and direct redox experimentation. Experimental characterization of these enzymes will provide valuable insight into the variance and mechanisms of chromate reduction by various *Thermoanaerobacter* strains.

## Abbreviations

Fe(III): Iron in the +3 oxidation state
Cr(VI): Chromium in the +6 oxidation state
Cr(III): Chromium in the +3 oxidation state
U(VI): Uranium in the +6 oxidation state
rRNA: Ribosomal ribonucleic acid
H_2_S: Hydrogen sulfide
K_2_CrO_4_: Potassium chromate
MnO_2_: Manganese dioxide
AQDS: 9,10-anthraquinone-2,6-disulfonic acid
NADH: Nicotinamide adenine dinucleotide
NAD(P)H: Nicotinamide adenine dinucleotide phosphate
